# Experiences of Older People Living at Home With Medication Use: A Qualitative Study From Lithuania

**DOI:** 10.7759/cureus.101671

**Published:** 2026-01-16

**Authors:** Agne Jakavonyte-Akstiniene, Rugile Ramanauskaite

**Affiliations:** 1 Department of Nursing, Institute of Health Sciences, Faculty of Medicine, Vilnius University, Vilnius, LTU; 2 Department of Nursing, Faculty of Health Sciences, Klaipėda University, Klaipėda, LTU

**Keywords:** elderly, experience, home, medication, qualitative research

## Abstract

Background

Older people face difficulties in managing various diseases that require the use of multiple medications. The most common challenges arise from adherence to medication regimens, polypharmacy, and insufficient knowledge about medications. To ensure safe medication use, assessing the problems associated with medication use in older people and the factors that contribute to them is necessary.

Methodology

The Consolidated Criteria for Reporting Qualitative Research (COREQ) checklist was used. A total of 10 older people participated in the study. The search for study participants was conducted using the snowball method. The data collection method chosen was a semi-structured interview, which was conducted according to a pre-determined plan. Content data analysis was applied.

Results

The total duration of all interviews analyzed was 3.21 hours (201 minutes). Eight women and two men participated in the study. The following four main themes were identified from the interviews: (a) the medications used by respondents and their side effects; (b) adherence to medication regimens; (c) respondents’ attitudes toward sharing personal medications with others; and (d) sources of information about medication use sought by study participants.

Conclusions

The study provides information that helps better understand older people’s experiences, challenges, and problems when taking medication. The most common issues related to medication use are memory impairment, lack of knowledge, and barriers in the healthcare system. Medication use is ensured by established habits, organization, and adherence to rules. The study provides insights into how to improve healthcare for older people.

## Introduction

The World Health Organization (WHO) reports that the number of older adults (aged 60 and above) is increasing worldwide [1]. The elderly population is growing faster than other age groups [2]. The aging process involves biological, psychological, and social changes [3]. This natural process continues throughout a person’s life, and the body’s cells lose their ability to regenerate, leading to degenerative processes that cause disease. As diseases increase, medication becomes integral to older people’s lives. When two or more chronic diseases are present, several different drugs are often prescribed, which can lead to polypharmacy [4]. Polypharmacy increases the risk of taking potentially inappropriate medications, which can lead to drug interactions that may worsen health [5]. People over the age of 65 who take five or more different medications face challenges. It is difficult for them to follow a complex medication regimen, remember when to take a particular medication, and some may decide to adjust their medication doses themselves without consulting their doctors [6].

The main factors influencing adherence to medication regimens are complex medication use, poor health literacy among patients, communication between patients and healthcare professionals, personal beliefs, and medication cost [7]. The results of one study showed that the most common problems related to medication use were polypharmacy (55.4%), challenges in administering medication (48.4%), low patient awareness of adverse drug reactions (47.3%), and problems with adherence to medication regimens (46.5%). The main factors influencing medication use were also revealed: forgetfulness, lack of awareness, and long waiting times at healthcare facilities [8]. In addition, a study conducted in Italy in 2022-2023 revealed that as many as 80% of the individuals (aged 60 and older) receiving home healthcare services take five or more medications daily. Healthcare professionals conducting reviews of the medications used identified the most commonly used (potentially inappropriate) medications as ibuprofen, furosemide, and pantoprazole. The irrational use of these medications may have negative consequences for health [9]. Another study revealed that the majority (85%) of older people had a very complex medication regimen, 92% were affected by polypharmacy, and almost all (98%) nursing home residents (98%) were taking at least one potentially unnecessary medication [10].

Further research has shown that older people have little knowledge about the effects of medicines and their ability to monitor their health. Instead of naming the medication they take, they identify them by their appearance, indications, duration of use, or dosage [11]. Failure to adhere to medication regimens can contribute to increased healthcare costs. Therefore, healthcare for older people requires greater attention [12]. An integrated approach to the patient is essential to improve quality of life and achieve optimal treatment outcomes. Successful treatment outcomes are closely linked to the patient’s ability to manage their medication independently or with the help of a healthcare professional [6]. Educating patients on pharmacological topics is critical because many older people find it difficult to name the medicines they take or their indications, indicating a low health literacy level. Poor knowledge of medications is associated with poor medication management [13].

Nurses play a significant role in independent medication management for older people who are typically multimorbid. Nurses are essential to the medical team, helping patients with their medication and building trust-based relationships with them [7]. Another study suggested that interventions that educate patients about their medications, strengthen motivation to adhere, build behavioral skills, and enhance treatment experiences may substantially improve short-term medication self-management capacity and adherence [14]. Lithuania lacks data on the specific experiences of older people when taking medication, as this is an under-researched area. Healthcare professionals should pay more attention to informing and educating patients to ensure safe medication use in the future. This study aims to analyze the experiences of older people living at home regarding medication use, focusing on how they understand, manage, and integrate their medicines into everyday life.

## Materials and methods

Study design

The Consolidated Criteria for Reporting Qualitative Research (COREQ) checklist [15] was used to conduct and present the study. The data collection method chosen was a semi-structured interview, which aimed to identify the experiences of older people when taking medication. The study was conducted between April and July 2025. The interviews were conducted in person (at the participants’ homes) and remotely (by telephone), depending on the participants’ abilities and preferences.

Recruitment of interviewees

The sample population consisted of older people living in Klaipėda County. The snowball sampling strategy was used to form the research sample. This method was helpful because it helped reach older adults with limited social contact. To find respondents, the article’s author (RR) used her personal social networks and posted an advertisement on them. A total of 10 respondents participated in the study. The selection criteria for the respondents were (1) aged 60 or older; (2) living in Lithuania and speaking Lithuanian; and (3) voluntarily agreeing to participate in the study. The criterion for exclusion from the study was refusal to participate.

Number of interviews needed for this study

Data saturation was sought to determine the experiences of older people when taking medication. As the responses of the ninth and tenth participants began to repeat themselves, when the new interviews did not yield any new categories or themes, and all of the main research questions already had sufficient data, it was decided not to expand the sample further and limit the number of participants to 10. Data saturation is one of the most critical criteria in qualitative research, used to determine when to stop collecting data. It is an important indicator of whether the sample is sufficient to analyze the phenomenon under study and whether the collected data reflects the diversity and depth of the subjects. The quality and validity of the study depend on data saturation [16]. The total interview time was 3 hours and 21 minutes.

Collection of quality data

The interview consisted of 10 questions divided into the following four parts: Part I consisted of three demographic questions that allowed the participants to be described. These questions aimed to identify the age, gender, and education level of the elderly participants. Part II consisted of two questions to assess the study participants’ health status, medications, and side effects [8,17,18]. Part III consisted of three questions investigating the adherence to and administration of medication regimens among study participants. Older people were asked whether they can adhere to their prescribed medication regimens, what should be changed, or improved. What problems do they encounter when taking medication, what causes these problems, what helps to ensure proper medication use, and how do older people distinguish between the medications they take? [8,12,19] When asking questions, respondents were asked to provide examples. Part IV consisted of two questions aimed at assessing the attitudes of survey participants toward sharing personal medications with others. They were also asked where they look for information about medicines; how often they talk to their doctor, nurse, or pharmacist about taking medicines; and what information they provide [7,8,20]. After the supervisor (AJA) training on conducting a semi-structured interview, a final-year student at Klaipėda University (RR) interviewed older people.

Before the interview, all participants were informed about the study’s purpose and the use of the data. During the face-to-face interviews (seven interviews), an informed consent form was signed. During the remote interviews (three interviews), verbal consent was obtained to participate in the study. The confidentiality of the study participants was ensured. The conversations between the researcher and the study participants were recorded. The transcription function in Microsoft Word 2022 was used to transcribe the interview. The automatic transcription was carefully reviewed several times and corrected manually to ensure its accuracy.

Data analysis

A content analysis was performed, which allowed the qualitative data obtained to be processed [21]. Qualitative content analysis focuses on understanding text content’s meaning, context, and trends [22]. In qualitative content analysis, coding is crucial to the research process. It involves assigning categories or codes to segments of the analyzed content (e.g., words, paragraphs, or pages) [23]. Two researchers coded the transcript. Coding was designed to be adapted to specific categorization [24]. The data of the study participants were coded using the codes R1, R2, R3, R4, R5, R6, R7, R8, R9, and R10. The data were divided into categories and subcategories. Each part was analyzed separately to assess the health status of older people, knowledge about the medicines they take and their side effects, and to reveal the adherence to and administration of the medication regimen of older adults, their attitude toward sharing medicines, and the sources of information about medication use sought by the study participants.

Ethical considerations

The Helsinki Declaration, an important document on biomedical research ethics, was followed to ensure ethical conduct of scientific research. The study was approved by the Ethics Committee of the Nursing Department, Faculty of Health Sciences, Klaipėda University, on April 17, 2025 (approval number: 46Sv-S-12). Each participant in this study provided verbal or written consent.

## Results

General characteristics of the patients

In total, 10 respondents participated in the study, including eight women and two men. The youngest woman was 60 years old, and the oldest was 76 years old. The youngest man was 61 years old, and the oldest was 68 years old (Table 1).

**Table 1:** Demographic data of the respondents.

No.	Age (years)	Gender	Education
R1	71	Female	Higher (university)
R2	64	Female	Higher (college)
R3	76	Female	Incomplete basic education
R4	76	Female	Higher (college)
R5	60	Female	Higher (university)
R6	61	Male	Higher (college)
R7	68	Male	Secondary education
R8	66	Female	Higher (college)
R9	69	Female	Higher (university)
R10	65	Female	Higher (university)

Four respondents (R1, R5, R9, R10) had higher education (university), four participants (R2, R4, R6, R8) had higher education (college), and one (R7) had secondary education. One respondent (R3) had an incomplete basic education (completed eighth grade).

Results of the content analysis

The following four main themes were identified from the interviews: (a) the medications used by respondents and their side effects; (b) adherence to medication regimens; (c) respondents’ attitudes toward sharing personal medications with others; and (d) sources of information about medication use sought by study participants.

Medications and Their Side Effects

The study aimed to find out what health problems respondents take medications for. The reasons for taking medications were divided into the following eight groups: (1) circulatory system diseases; (2) endocrine system diseases; (3) mental disorders; (4) diseases of the digestive system; (5) diseases of the urinary system; (6) diseases of the musculoskeletal system; (7) diseases of the nervous system; and (8) diseases of the eye and its adnexa.

Most respondents took medication for circulatory system diseases. This reason was most frequently mentioned among all responses, with eight respondents indicating that they took medication for high blood pressure (hypertension):

“I take medication for high blood pressure, since I was 50 - Triplixam - it is for high blood pressure” (R1). “And because of high blood pressure … so that it does not cause problems, I take these medications, they lower my blood pressure” (R2). “I take Heart Drops, I take Aspirin, <…> for blood pressure” (R3). “I take it for blood pressure <…>” (R4). “At first, I blamed stress. I went to the doctor, he looked at me and said - You need medication. I’ve been taking it ever since” (R5). “I have hypertension, so I take medication for my blood pressure in the mornings” (R6). “I have hypertension, so I take medication in the morning and in the evening <…>” (R7). “Every day, for blood pressure, high blood pressure <…> Nebilet is in the evening, and I won’t tell you the morning dose because it was just prescribed to me” (R10).

Cardiovascular diseases were among the most common health problems for which older people took medication, most commonly antihypertensive and antiplatelet drugs. Some patients tended to take antiplatelet drugs, i.e., aspirin, most often for the prevention of cardiovascular disease:

“Well, sometimes I take half an Aspirin in the evening, sometimes I drink it” (R3). “<…> and I definitely need Aspirin, because it thins the blood” (R6). “<…> I take it as a preventive measure, because it … thins the blood - Cardio Aspirin” (R7).

A healthcare professional must supervise aspirin use, as self-medication can cause unwanted side effects.

Three study participants had diabetes:

“For diabetes, I take one tablet in the morning and one in the evening. The doctor said I don’t need insulin yet, which makes me very happy” (R2). “Metformin, for diabetes <…>” (R4). “<…> and I have type 2 diabetes - I take two tablets in the morning and two in the evening <…>” (R7).

Some respondents were taking medication for high cholesterol:

“Plus, I have high cholesterol, so I also take medication-Roswera” (R1). “I still have some problems with cholesterol, I take medication for cholesterol too…” (R6). “<…> I have cholesterol … well, it’s high, but I control it <…>” (R7).

One participant in the study indicated that she took medication for chronic thyroid disease: “My thyroid, about ten years ago. They said I had … Hashimoto’s, I think it’s called. So now I take a pill every morning. I’m used to it, I’m used to it. Like brushing my teeth - I don’t even think about it anymore…” (R5).

Some respondents indicated that they have mental health problems for which they take medication: “I take sedatives to help me sleep. I don’t know what they’re called, something like ‘Zol…’, I can’t remember exactly, but I try not to take them every day” (R2). “These are tranquilizers for mental health: Sertralin, Mirtazapin, some to take in the morning, others in the evening” (R4). “I am being treated at a mental health center because I had very severe depression, and I take two medications from there” (R8). Thus, three respondents had insomnia or depression.

One participant in the study complained of digestive system disorders:

“Nolpaza, for my stomach, so I don’t get heartburn. I take Nolpaza so I don’t” (R3).

Two study participants also reported having urinary tract disorders for which they take medication:

“Just that … I use it for bladder inflammation … How can I put it … a disorder” (R4). “And the third medication, <…> for a bladder damaged by multiple sclerosis. I take Zavesin” (R8).

Two participants indicated that they had muscle and skeletal disorders for which they used rubbing medicines: “I rub my legs with camphor spirit. <…> Voltaren, Diclac ointment” (R3). Participant R4 also indicated that he rubbed his legs.

One participant suffered from a nervous system disease: “I have multiple sclerosis, the left side of my body is affected, it is difficult to walk with one supporting leg, and I sway from side to side, which makes me unstable. These medications are not taken orally, but are administered twice a year to suppress the immune system” (R8).

Respondent R1 suffered from diseases of the eye and its accessory organs:

“And I have a genetic disease - glaucoma, which is awful, really awful, truly awful.” “<…> I also take some medication, from the beginning of the pandemic until this very moment, and they prescribe it every two months, sometimes every three months. Eye drops” (R10).

When analyzing the data, it can be seen that respondents took various dietary supplements and vitamins:

“Now they prescribed it at the hospital, told me to take probiotics, so they gave me a fourteen-day course at the pharmacy” (R2). “I drink fish oil. I drank … I have not finished it yet, but I drank the calcium, magnesium, and zinc” (R3).

Adverse drug reactions were identified based on general knowledge and individual cases based on personal experience. When analyzing the data, it can be seen how one of the study participants responded by naming the most common side effects of drugs:

“Oh, there are so many side effects: nausea, headaches, diarrhea … oh, everything” (R2). He later added specific examples from his personal experience, linking the drug and its possible side effects: “Well, for example, with Metformin [he has difficulty pronouncing the name], I know that this medicine can cause nausea, especially if you take it on an empty stomach … That’s what happened to me at first - I felt nauseous, but then, when I started taking it after meals, everything was fine” (R2).

One participant in the study also provided an example:

“There was a time when my thyroid gland was prescribed a slightly excessive dose, and I began to feel anxious, and my heart was pounding” (R5).

Another participant also shared their experience:

“I encountered this … and personally … it got worse for me … awful, and that’s bad with multiple sclerosis <…>. Moreover, this and … well, while taking these medications, I didn’t understand why my bladder was getting worse and worse. <…> and then, doctor, there was muscle tension” (R8).

One study participant talked about the side effects that occurred after starting to take the newly prescribed medication:

“There are side effects, I’ve never had swollen legs in my life, but now, my legs swell up, and maybe it’s because this medication, the one I had before, always had to be taken with twenty-five milligrams of a diuretic, some kind of additive, and the compound medication was taken in the morning, and now, maybe I didn’t pay attention, maybe I’m looking at something, on the second day…” (R10).

It is important to note that one participant in the study stated that he was unaware of any possible adverse effects of the medication:

“I don’t know of any adverse effects. I don’t know of any such effects. It’s always the same, it’s always the same. There are no such effects” (R4). When talking about the adverse effects of drugs, informants relied on their individual experience and knowledge.

Compliance With the Medication Schedule

During the survey, respondents were asked how often they complied with their medication schedule. Most older adults replied that they managed to comply with their medication schedule:

“Well, I manage to do what I have to… <…>. Well, how could you forget if it hurts?” (R3). “I take enough and I take them conscientiously” (R4). “Well, basically, I usually manage to comply. That’s how I am - I try to follow the rules…” (R5).

One participant managed to stick to the regimen and, to ensure continuity of treatment, took her medication with her when she left home:

“I can’t forget. Because eye drops are … If I’m not coming back in the evening, I stay overnight, so I keep my medication in my bag, as well as my blood pressure medication, and I take it in the morning” (R1).

Other respondents also adhered to a successful medication regimen:

“No, that doesn’t happen, I’m a very responsible person. <…> No, I manage everything very well with my medication” (R8).

During the interview, it became clear that one participant in the study had been careless about taking her medication in her youth, but had begun to take a responsible approach to the process in her older age:

“At first, I was very superficial about it, like, I’m going to the pool this week, they told me to take it so that nothing happens - I’ll take it. I’ll take it, I won’t take it, I’ll take it, I won’t. However, since I’m mature, I’m afraid I won’t be able to control that pressure <…>” (R10). Thus, six participants in the study managed to adhere to their medication regimen.

One participant stated that she usually managed to adhere to the regimen, but when her environment changed, or her health improved, she forgot to take her medication:

“For diabetes, I take it in the morning and evening, after meals. However, when there is a celebration or I go out to visit someone, my eating schedule gets mixed up, and I can completely forget to take that medication” (R2).

Another participant pointed out that the medication regimen process should be improved:

“Well … we could definitely improve that. I don’t follow everything 100%. There are days when I forget. Especially in the mornings, because I wake up with a clear head, as they say, and, well, I forget, but everything happens, everything happens” (R6).

Another participant also faced the problem of forgetfulness:

“<…> well, I don’t know, sometimes I forget, but it’s better not to take it if you don’t remember” (R7). Thus, three participants in the study claimed that they encountered difficulties and challenges in adhering to their medication regimen.

When analyzing the adherence to and administration of medication regimens among older people, it was essential to identify the problems encountered by the study participants when taking medication. The problems were divided into the following three groups: (1) problems related to the study participants’ lack of knowledge about medication use; (2) barriers in the healthcare system; and (3) unclear medication information leaflets.

The study participants’ responses reflected insufficient knowledge about medication use. One older adult stated that they did not know enough about newly prescribed medications:

“New medications are harder to understand; you have to read and ask questions, but you don’t always ask. Sometimes I feel that I don’t understand enough about the use of medicines <…>” (R2).

Another participant claimed that the problem was not knowing whether prescribed medications can be taken with food:

“Sometimes the problem is not knowing what you can mix with them and what you can’t. For example, I like to drink coffee in the morning. However, a doctor once told me that with thyroid pills, I should only drink water, no coffee, no breakfast right away” (R5).

Another participant indicated that she did not know how to take dietary supplements correctly:

“I drink fish oil now. <…> I drink it when I remember. I don’t know how to take it” (R3).

Another participant in the study stated that she often had doubts about when exactly to take her medication:

“<…> it says morning and evening, but it’s medicine, so I take it. What do I know? One day, in the morning, I wake up at maybe ten o’clock. I take it to be morning, but why isn’t it morning? Another time, when I go to work, I leave at 7:15, but I take it at around 7:00, and it’s still morning” (R10). Thus, the respondents’ insufficient knowledge about medication use is a pressing issue.

Four participants in the study identified obstacles in the healthcare system as a problem arising from medication use. An older adult stated that they consciously avoid going to the doctor because they do not want to bother them; according to the respondent, they are always busy:

"I don’t want to bother doctors and constantly ask questions because they are always in a hurry and don’t have the time” (R2).

The doctor did not prescribe specific medications, so the respondent resorted to self-medication: “They don’t give me any medication for my legs. I buy Diclac or camphor spirit myself, and that’s it. I go and buy it myself, without the doctor’s knowledge” (R4).

During the interviews, it became clear that the study participants had problems reading the information leaflets that came with the medicines.

One participant in the study indicated that they encountered difficulties when reading the medication information leaflet due to the excessive amount of information:

“<…> we … only read about how to use it, when to use it, how much to use, we try … because there are so many sources. Moreover, so many points may be undesirable for ten percent of a hundred or two hundred people” (R10).

To better understand the experiences of older people in adhering to medication regimens, study participants were asked what helps them ensure medication adherence and what aids they use to ensure medication adherence. Some of the study participants did not use any aids when taking medication, while others chose to rely on habits formed over a long period of medication use:

“Well, you know, habit plays a big role here … When you’ve been taking the same medication for years, your body reminds you that you need to take it. You open your eyes, take your thyroid pill, and your blood pressure pill after breakfast. It’s just in your blood. You feel responsible. So, I usually do it without any notes, but out of habit” (R5).

Others remembered that it was time to take their medication:

“I still remember” (R3). Another participant in the study remembered and reminded her relatives: “And my medication is … I don’t like them, but I remember them; my brain still works, apparently?” (R10).

A different opinion was also expressed.

One participant stated that a daily routine helps him take his medication:

“Well, no, it’s the same thing, you stick to a routine. I prepare my medication the night before so that I’m sure to see it” (R2).

Another participant in the study used a box in which medicines are sorted according to the days of the week:

“Or I have a medicine box. Several compartments, there are morning ones, evening ones, for blood pressure, for diabetes. <…> as many as fit, those medicines, from seven compartments” (R4).

Another participant in the study used a mobile phone to help with the medication process: “Well, I think that in the 21st century, my main helper is my phone, which helps me remember all the times when I need to take something” (R7).

The next part of the question aimed to determine how the respondents separated the medications they take. Two respondents replied that they have no problems separating the medicines they take:

“Well, after so much time, you just remember. So many years ago. I took those medications when I broke my leg. It’s been about 5-6 years for high blood pressure. Moreover, I’ve been taking Nolpaza for about 8 years for heartburn. <…> I’ve been taking Diclac for a long time” (R3). “I don’t confuse them” (R5).

One respondent said that he identified the medications he takes by the packaging or the size of the tablets:

“Well, I distinguish those medications by the box or the tablets themselves. The diabetes ones are white and bigger. Zolpidem is in a small, oblong box. The blood pressure medication is in another box with a triangle on it” (R2). According to the respondents’ answers, it can be seen that older people do not have difficulty distinguishing between medicines, and one of the respondents distinguished them by visual characteristics.

The Attitudes of Study Participants Toward Sharing Personal Medications With Others

Three study participants responded that they were against sharing medications:

“That’s nonsense. What works for one person may not work for another. How can you offer your illness to someone else? No way, no how” (R1). “I never share. <…> No. I don’t ask anyone for medication, and no one asks me for medication” (R3). “Well, I don’t really offer anyone medication that isn’t suitable for them. There have been occasions when someone has offered me something and told me what to take, but I prefer to follow the doctor’s instructions” (R6).

Another participant was against sharing medication with others and also gave an example from their immediate environment:

“No, no. It has happened; I saw it myself - one sister comes to visit another sister. She says, ‘I left my medicine’. The other one says, ‘Take mine’” (R7).

Two participants in the study were also against sharing personal medication, and they gave an example of people who do this:

“The bad thing is that older people, I know, share, my neighbor had some medicine left over, give it to me, maybe it will help, for blood pressure, oh, I almost fell off my chair” (R8). “I know that after the funeral, I was offered all kinds of sedatives and there were many advisors … I just politely declined” (R9).

Four participants in the study tended to share their personal medications with others. One participant rarely found herself in such a situation, but when necessary, she shared her medications with her loved ones:

“Well, in such situations, it doesn’t happen often, but when it does, it’s mostly with acquaintances, neighbors, or relatives who feel unwell. For example, they say they have a headache, so I give them ibuprofen” (R2).

One participant in the study also stated that he shared medication with family members and sometimes took prescription medication that he got from a loved one:

“I take some, sometimes. <…> I’ll tell you, recently. My mom. Trifas, it’s prescribed for her, so to speak. Mom, I say, Jesus, look what’s happening to me in the evening. You know, she says, right away, take Trifas from me and run, it’s no big deal. In fact, it’s been more than one morning when I didn’t have to go to work, it was a day off, and I used it. I share” (R10).

Another participant in the study also indicated that they shared over-the-counter medications:

“Well, I share simple medications, if I use them, for acidity, so that I can give them to others, but I don’t share the medications that I mainly use myself” (R4).

Another participant in the study (R5) expressed a similar opinion:

“Anything can happen in life. I usually keep some Paracetamol on hand.”

Information Sources on Medication Use

When conducting the study, finding where older people looked for information about their medications was essential. An analysis of the responses of older people showed that three main groups could be identified: (1) the internet, (2) healthcare professionals, and (3) medication instructions.

The study participants chose to obtain information from electronic sources:

“I spend much time on Facebook. If something is unclear to me, I look it up there, maybe even too much, but I don’t post anything myself. But I read it” (R1). “Well, I go to Google, search the internet, often visit forums where other people talk about how they feel after taking the medicine. I read about this Metformin, which has a terrible effect on the kidneys of some people” (R2).

One participant in the study searched for information about medications based on other people’s experiences on the internet: “<…> I participate in an international Facebook group, there is a chat, only about multiple sclerosis, and thanks to it … I discovered this Zavesin because I asked people to write what medications they take for urinary problems, as mine no longer help me after fifteen years of use, and they used to prescribe reimbursable ones” (R8).

Another participant in the study was interested in the ingredients of medicines:

“Yes. The Internet is all-powerful. On the Internet, you can find out more details, and if you enter the active ingredient, you can really find some good information there” (R10). Four participants in the study searched for information about medicines in the digital space.

One participant indicated that they obtained information about medications by listening to radio programs:

“I’ll tell you something else, I watch ‘Miego DNR” (Sleep DNA), I listen to it. <…> on the radio, on Sundays” (R7).

One participant also read the press to find out about his medications:

“Well, I even … for example, ophthalmology … magazines, or something else, are almost like lifestyle magazines to me” (R10).

Most of the study participants got their information about medicines from healthcare professionals:

“And when I go to my doctor, I haven’t even finished telling her, [name], and she already says, You want to know everything. Moreover, she’s happy to tell me, kid, and she writes it down. <…> Pharmacists advise that if you’re buying antibiotics, you should also buy some good bacteria” (R1).

Another participant in the study also emphasized that they talked to their doctor about medication, although they consulted with the pharmacist more often:

“Well, I talk to my doctor, but not that often. I often talk to the pharmacist when I buy medication. I ask: ‘What about food - is it okay?’ <…> Well, about those sedatives, the doctor told me more, that you can get used to them, that you can’t use them together with strong drinks” (R2).

Another participant in the study indicated that they always consult with their physician, and sometimes with their nurse: “I always ask the doctor. I am familiar with her, as I have been seeing her for many years-she always explains things in simple terms. I also talk to the pharmacists sometimes. <…> Nurses are somehow closer to people. There is one nurse like that in our clinic - when she takes my blood or measures my blood pressure, she always asks how I am doing with the medication” (R5).

Another participant in the study made a similar statement:

“Well, I always go to my family doctor because I trust her. She knows all my health problems and can also do tests to select the right medication and possible medications for me” (R6).

Many participants also indicate that they consult pharmacists about medications: “I ask at the pharmacy” (R3). “Well, I go to the pharmacy <…>. You have to communicate, not shut yourself off” (R7).

Another participant in the study gave an example:

“I’m in line at the pharmacy. For example, I’ve had a sore throat for two weeks, so I went to the pharmacy and asked, ‘Help me, what should I do?’” (R8).

Several participants in the study also looked for information about medicines in the instructions for use:

“Yes, where it’s written on the inside” (R3). “In the instructions for the medicine” (R4).

The study participants provided various sources where they look for information about medicines and health: medicine instructions, radio programs, and magazines.

## Discussion

Problems related to the safe use of medicines are relevant in many countries worldwide [25-26]. In our study, eight participants indicated that they suffered from arterial hypertension. Studies have emphasized that arterial hypertension is common among older people [27-29]. Our study showed that a small proportion of respondents took aspirin to prevent cardiovascular disease. Scientific literature reports that the positive effects of aspirin have been observed in people under the age of 70 who are at high risk of cardiovascular disease and do not have an increased risk of bleeding [30]. However, as long-term use increases the risk of bleeding, more attention should be paid to reducing the inappropriate use of aspirin among older people [31,32].

Our study identified the difficulties that respondents encounter when reading and learning about the adverse effects of drugs. Several study participants shared their personal experiences related to the adverse effects of drugs they have experienced. Christopher et al. (2023) conducted a study that revealed a lack of knowledge among older people (aged 60 and above) about the side effects of medicines. A total of 393 participants took part in the study. Of all respondents, 52.7% (n = 207) were aware of the side effects of the medicines they were taking, while one-third of respondents (33.6%, n = 132) confirmed that they were unaware of the possible side effects of the drugs [8].

Health literacy refers to a person’s ability to understand, analyze, and apply health information to make informed decisions [7]. Respondents who participated in our survey most often obtained information about drugs from healthcare professionals (doctors and pharmacists) and the internet (Facebook groups and internet forums), with one respondent indicating that they use the artificial intelligence tool ChatGPT. In comparison, few study participants analyzed the medication information leaflet. Some respondents revealed that they lack a deeper understanding of medication use, but feel uncomfortable asking doctors questions because they do not want to bother them. Dijkstra et al. (2022) reported that successful treatment outcomes are based on the ability to independently manage medications, which requires specific knowledge, especially when taking multiple medications [6].

In our study, six participants adhered to their medication regimens as prescribed in their treatment plans. The study revealed that some respondents encounter various obstacles in adhering to their medication regimen. The problems mentioned included forgetfulness, missing doses due to changes in environment or meal times, and one respondent practiced taking medication based on how she felt at the time (she took medication when she felt bad and did not take it when she felt well). Christopher et al. (2023) also claimed that the main factors influencing medication use are forgetfulness and lack of awareness. The findings show that about 50% of older people face problems with medication use, with difficulties arising from medication administration and adherence to the regimen [8].

The most common problems associated with medication use are polypharmacy, low awareness of adverse drug reactions, adherence to medication regimens, and difficulties in accessing primary healthcare services [8,33,34]. In this study, the most common responses were lack of knowledge, obstacles related to the healthcare system, and forgetfulness. Respondents stated that they find it more challenging to understand newly prescribed medications, lack knowledge about taking prescribed medicines with food, and when exactly to take them.

Our study found that nearly half of the participants had a favorable opinion about sharing medicines with others. Respondents indicated that they found sharing medicines within their family circle acceptable. Dawson et al. (2024) emphasized that sharing prescription drugs with others may increase the risk of side effects, delay seeking medical attention, or worsen the condition. Analgesics, antibiotics, and allergy medications are most commonly shared [35]. Furthermore, Baracaldo-Santamaría et al. (2022) reported that medicines shared among family or friends are a global health problem prevalent in developing countries, where this trend is becoming increasingly common due to poor access to healthcare, a shortage of medical personnel, or extreme situations [36].

Strengths and limitations

This study had many strengths. The qualitative methodology enabled a deeper understanding of the experiences of older people, utilizing semi-structured interviews that captured personal perspectives and contextual information. Second, the study involved men and women aged 60 and older, all of whom were able to provide meaningful information about their medication use. Thus, this diversity likely added strength and rigor to our qualitative data. Third, this is the first qualitative study conducted in Lithuania that focuses on the experiences of older people living at home about medication use. The study results can help healthcare professionals and policymakers develop recommendations for improving the quality of care for older people.

Several limitations of this study should be noted. First, as the study was conducted in a specific geographical area (Klaipėda County, Lithuania), the applicability of its results to other regions is limited. Second, the study included 10 older individuals who met the requirements for qualitative research; however, the study’s results cannot be generalized to the entire population of older people. Additionally, a long-term observational study would provide additional information on this topic. The significant gender imbalance, eight women and only two men, may have a substantial impact on the results, especially given the potential gender differences in help-seeking behavior and attitudes toward medication management. Relying on snowball sampling may result in selection bias, for example, as participants tend to recommend people who are similar to themselves, which can lead to a sample that is more homogeneous than the actual population. Additionally, snowball sampling is more likely to include individuals who are more active, accessible, and willing to participate in studies, while less visible or vulnerable groups may be underrepresented.

## Conclusions

Most study participants took medication for cardiovascular diseases, such as aspirin. Medication was also taken for pain, digestive, urinary, nervous, eye, muscular and skeletal, and mental health disorders. A small proportion of respondents mentioned the undesirable effects of the medications, the names of the medications they were taking, and their purpose. Some respondents carefully followed the prescribed treatment plan and medication regimen, but others experienced difficulties. The most common problems related to medication use were memory problems, lack of knowledge (e.g., did not know whether they can take medication with food, or what the exact time should be if it is indicated to take it in the morning), and obstacles in the healthcare system (e.g., did not want to bother doctors, or doctors did not prescribe new medications). The study participants did not encounter issues identifying the medications they take, because they used medicine boxes and had been taking the same drugs for several years and were familiar with them. Some older people considered sharing personal medications with others, e.g., ibuprofen or paracetamol.
